# Case Report: Duodenal Carcinoma in a 40-Year-Old Asian Man With Cowden Syndrome

**DOI:** 10.3389/fsurg.2022.935048

**Published:** 2022-07-12

**Authors:** Mingkun Zhao, Xiaohan Lin, Yuan Fang, Aobo Zhuang, Hanxing Tong, Weiqi Lu, Yuhong Zhou, Yong Zhang

**Affiliations:** ^1^ Department of General Surgery, South Hospital of the Zhongshan Hospital/Shanghai Public Health Clinical Center, Fudan University, Shanghai, China; ^2^ School of Clinical Medicine, Hangzhou Normal University, Hangzhou, China; ^3^ Department of General Surgery, Zhongshan Hospital, Fudan University, Shanghai, China; ^4^ Department of Medical Oncology, Zhongshan Hospital, Fudan University, Shanghai, China

**Keywords:** Cowden syndrome, duodenal carcinoma, Whipple, Asian, PTEN

## Abstract

**Introduction:**

Cowden syndrome is a rare autosomal dominant genetic disease associated with PTEN mutation and is mainly shown as systemic multisystem lesions. The incidence of adenocarcinoma of the duodenum with Cowden syndrome in Asian males is rare. We hereby describe the diagnosis, treatment, and prognosis of a patient with duodenal carcinoma and Cowden syndrome.

**Case Description:**

A 40-year-old Chinese man was hospitalized because of gastrointestinal hemorrhage and anemia due to infiltrating adenocarcinoma of the descending part of the duodenum. He also had typical signs of Cowden syndrome, such as multiple polyps of the gastrointestinal tract, macrocephaly, papilloma of the tongue, soles hyperkeratosis, and melanosis spots. After the pancreaticoduodenectomy (classic Whipple), the lesions revealed the presence of hamartomatoid polyps, and some of them mutated into non-mucinous adenocarcinoma (80%) and mucinous adenocarcinoma (20%). Further investigation showed a lack of PTEN protein expression in the duodenal neoplasm, and genetic analysis showed the mutation of p.E242fs in PTEN. The patient was followed up for 1 year. There was no appearance of recurrence or distant metastasis.

**Conclusion:**

It is suggested that we should pay more attention to the differential diagnosis of duodenal carcinoma combined with gastrointestinal polyps. If multiple gastrointestinal polyps with gastrointestinal bleeding are encountered, Cowden syndrome should be considered, and timely diagnosis and treatment should be implemented.

## Introduction

Cowden syndrome (CS) has been identified as a rare autosomal dominant genetic disease associated with *PTEN* (located at the 10q22–23 locus) mutation (1, 2). The first case of CS was reported in 1963 (3). The *PTEN* gene is a tumor suppressor gene that encodes an essential negative regulator of the phosphatidylinositol-3-kinase signaling cascade (4–6). The mutant outcome of *PTEN* is characterized by hamartoma and an increased lifetime risk of malignancies in patients with Cowden syndrome (7). In addition, most patients with CS tend to have macrocephaly and widely differentiated skin lesions. Specific mucocutaneous manifestations of CS include trichilemmomas (especially on the face), acrokeratosis, mucocutaneous neuromas, oral papillomas, and melanosis spots on the glans of the penis (8). Except for genetic testing, the diagnosis depends typically on clinical criteria. The criteria were updated in 2019 by the National Comprehensive Cancer Network^®^ (NCNN^®^) (9, 10).

We present the case of a 40-year-old man with Cowden syndrome who had 15 years of gastrointestinal bleeding history. The physical and auxiliary examination revealed that he developed a representative appearance of dermatology and polyps in the stomach and colorectum, combined with duodenum adenocarcinoma. Further genetic testing showed an inactivating mutation in the *PTEN* gene. His adolescence was normal, his parents were alive and in good health, he had no siblings, and he had not married.

## Case Report

In 2021, a 40-year-old male patient came for consultation. He had chronically recurrent gastrointestinal bleeding for 15 years, developed malignant lesions of the descending part of the duodenum, and then underwent pancreaticoduodenectomy in our hospital. The patient has lost 15 kg of weight since the onset in September 2020. Moreover, he had no notable family medical history.

**Figure 1:**
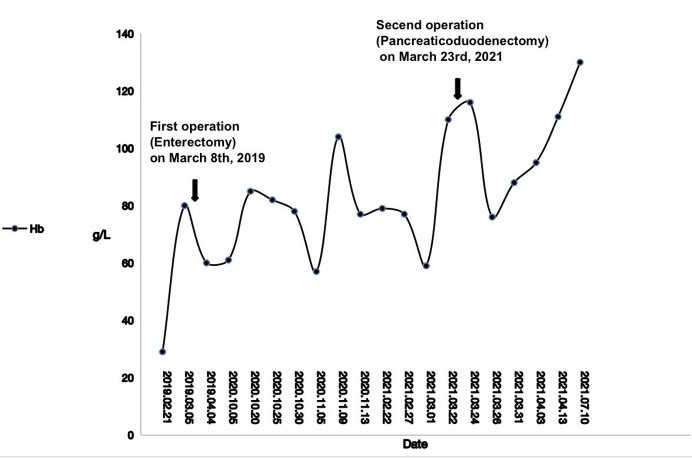
Changes in hemoglobin values between two surgeries (February 2, 2019 to July 10, 2021).

The patient was admitted to the local hospital on March 8, 2019, and operated on a partial enterectomy with a 150 cm intestine because the enteroscopy found bleeding in a part of the intestine. However, the patient was still under anemia after the operation (Figure 1). After then, the patient suffered from jaundice and incomplete ileus with the feeling of ventosity and nausea in October 2020. The local hospital implemented PTCD to improve jaundice and treat fasting, nasogastric drainage, and total parenteral nutrition for incomplete ileus. However, they did not find a solution to handle the bleeding event. Therefore, the patient came to our hospital for further medical consultation. Tracing the history, we found that the patient had anemia before he was 16 and was given blood transfusion therapy. This situation recurred 3–4 times before.

**Figure 2:**
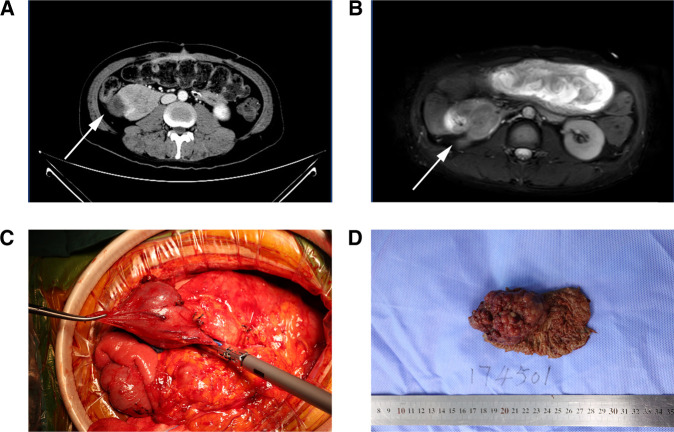
(**A**,**B**) CTV (left) and MRI (T2WI) (right) revealed a space-occupying lesion located in the descending part of the duodenum, (**C**) huge tumor of the descending part of the duodenum was seen during the operation, (**D**) gross specimen of the dissected tumor.

We did regular pre-operation examinations after the patient was admitted to our hospital. Enhanced computer tomography showed dilatation of the duodenum and stomach, and there was a space-occupying lesion that tended to be malignant. Magnetic resonance imaging revealed duodenal malignancy, slight dilatation of the bile duct, and fatty liver (Figure 2). Obviously, the gastrointestinal obstruction was caused by the occupying lesion of the descending part of the duodenum.

**Figure 3:**
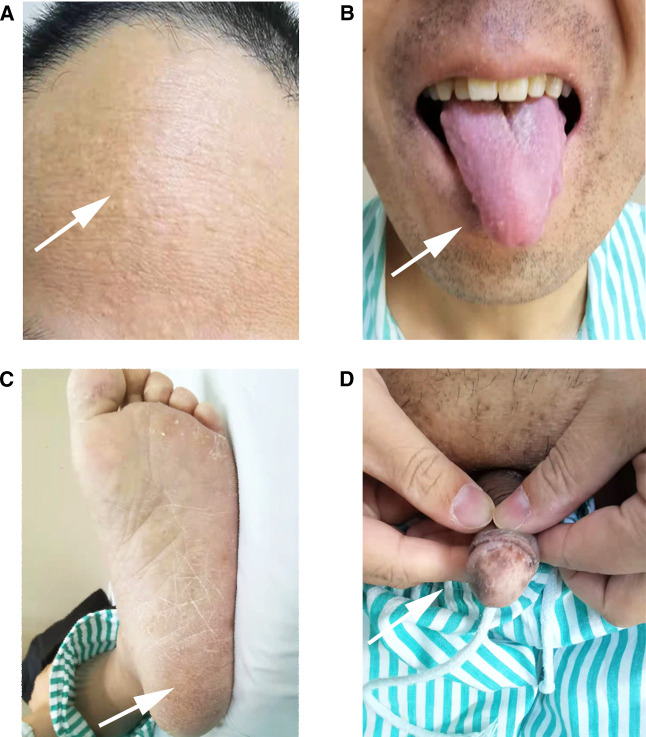
(**A**) Miliary papules were scattered on the forehead and face, (**B**) papilloma can be seen on the tongue, (**C**) some excessive keratinization can be seen on the soles of the foot, (**D**) melaniosis spots were on the glans of the penis.

Upper gastrointestinal endoscopy revealed multiple diffused polyps located in the stomach and the duodenum, and colonoscopy showed the same extent polyps of varying sizes localized in the rectosigmoid region. The onset of small intestinal malignant tumors combined with upper and lower gastrointestinal tract polyps might suggest MUTYH-associated polyposis syndrome (MAP) or Lynch syndrome (11, 12). Nevertheless, further physical examination revealed that the patient had macrocephaly, and dermatologic examination was remarkable (Figure 3). Miliary papules were scattered on the face, especially on the forehead. Also, there was papilloma of the tongue, hyperkeratosis of soles, and melanosis spots on the glans of the penis. Therefore, we considered the possibility of other diseases such as Cowden syndrome and P-J syndrome and performed a radical pancreaticoduodenectomy (classic Whipple) to remove the malignant lesion in the duodenum on March 23, 2021 (13, 14).

**Figure 4:**
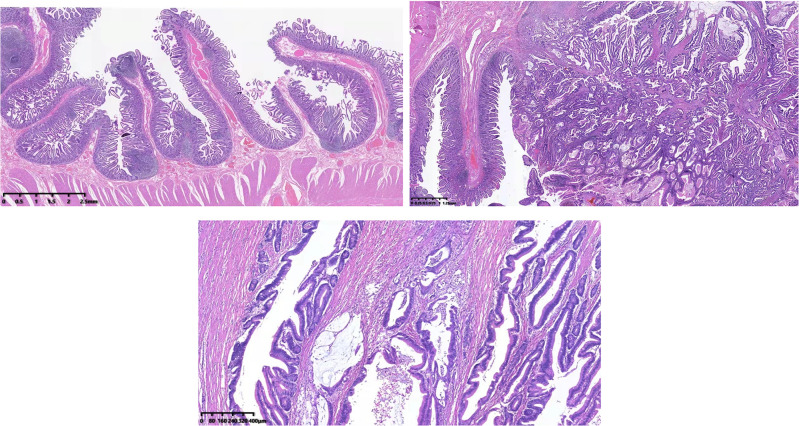
Hematoxylin–eosin staining showed that adenocarcinoma cells formed glands or adenoid structures with different sizes, shapes, and irregular arrangements; the carcinoma tissue had infiltrated into the subserous layer.

The gross pathology showed a 6 × 5 × 4 cm cauliflower-like protrusion lesion (Figure 2). The cut surface of the tumor was pale, soft, and fragile and visually infiltrated the muscular layer of the duodenum. Diffuse hyperplastic grayish-brown polyps could be seen in the entire intestinal wall, ranging from 1 to 4 mm. The common bile duct was dilated, and the pancreas was undisturbed. Microscopic pathology (Figure 4) reported that the cauliflower-like elevated tumor of the duodenum was considered a hamartomatous polyp, with malignancy in some areas (80% non-mucinous adenocarcinoma, 20% mucinous adenocarcinoma). Also, the carcinoma tissue had infiltrated into the subserous layer. Other polyps were described as polypoid hyperplasia of mucosal glands. No metastasis was observed in perigastric lymph nodes and peripancreatic lymph nodes. In addition, it is suggested that the mesentery nodules were hemangioma. Immunohistochemical results showed Ki-67(40% positive), CDX2(+), MSH2(+), MSH6(+), PMS2(+), CK20(+), STK11(+/−), PTEN(expression deletion), Her-2(weak +), SATB2(little +), D2–40(no apparent lymphatic tumor thrombus was found), and p52(DO7) (70%+); special staining showed no definite venous invasion of elastic fibers. Immunohistochemical results showed that PTEN protein expression was lost in the tumor and surrounding mucosal tissues, which may be an important factor in tumorigenesis. However, MLH1 and STK11 proteins were normally expressed. Therefore, we believed Cowden syndrome was the most likely diagnosis. At the suggestion of the pathologist, further NGS gene testing was carried out.

Gene testing of the tissue samples suggested multiple germline gene mutations. A frameshift mutation was detected in the *PTEN* gene, leading to amino acid p.E242fs alternation. We refined the localization to position 89717695 of chr10, which might lead to a premature terminator in the new reading frame, resulting in protein dysfunction and having pathogenic significance. According to the large population databases (gnomAD, E×AC, 1,000 genome, ESP6500, etc.), this was a rare mutation that has never been discovered before. Partial genetic mutations of the *PTEN* gene are related to PTEN hamartoma tumor syndrome/Cowden syndrome. This autosomal dominant genetic disease is associated with an increased risk of breast cancer and thyroid disease and may increase the risk of endometrial cancer and central nervous system tumors. On chr13, missense mutation occurred in two-locus—32906558 and 142274875, causing P.C315S of the *BRCA2* gene and p.T729A of ATR change. Missense mutations occurred in *PALB2* and *MC1R* gene on chr16, causing p.Q1114R and p.A166G changes. All mutations of these germline genes were heterozygous mutants, and their clinical significance for CS has not been clarified except for PTEN. Four critical tumor driver gene mutations were detected in the patient. Tumor mutational burden was 2.20 muts/Mb, and microsatellite instability was detected as microsatellite stable. The mutation in exon 2 of KRAS changed P.G12A inactivated GTPase, leading to the accumulation of tumor-associated KRAS in the active GTP-bound conformation. That possibly promoted the development of malignant tumors (15). Currently, there are drugs targeting KRAS gene mutations in the market. Nevertheless, the detection of KRAS mutation also suggested that patients with colorectal cancer may be resistant to Cetuximab(A) and Panitumumab(A). These discoveries can be expected to guide the follow-up treatment. The other three mutations, resulting in p.R714H, p.A4083P, and p.D351G alternations, were detected in exon 11 of *CREBBP*, exon 48 of *KMT2C*, and exon 9 of *SMAD4*. However, the clinical significance of these mutant genes is unknown. PD-L1 immunohistochemical staining score is as follows: TC ≥ 1% and IC+:2%; the result was positive.

Based on that evidence, the diagnosis of Cowden syndrome is confirmed. Since there were no indications such as regional lymph node metastasis, postoperative adjuvant chemotherapy was not performed. The patient was followed up for 1 year, and there was no postoperative obstruction of bilioenteric anastomotic stenosis. Gastrointestinal bleeding did not recur, and the anemia had been corrected. Repeated reexamination of tumor markers did not suggest recurrence. All these suggest a good prognosis.

## Discussion

Cowden syndrome (CS) is considered a component of the “PTEN hamartoma tumor syndrome” (PHTS) because the mutation in the *PTEN* gene was carried by most patients (80%) with CS (16, 17). Because *PTEN* as the gene responsible for CS had been identified, the estimated incidence rises from 1/1,000,000 to 1/200,000. Multiple hamartomas of ectodermal, mesodermal, and endodermal origin are typical characteristics of CS (18). However, the cognition and awareness of this syndrome disease are still at a low level, which means a lack of specific treatments. The gastrointestinal tract involvement in CS patients tended to be very frequent. According to the literature review of Innella et al., colon polyps were detected in 91.7% of the PHTS patients who were undergoing endoscopy, while extra-colonic polyps were detected in 58.5%. Moreover, the most frequent histology is hamartomas (65.8%) (17). In addition, 90%–93% of CS patients with *PTEN* mutations have polyps after undergoing colonoscopy (19).

As far as CS is a hamartoma syndrome, polyps in patients with CS have long been considered to have low, malignant potential. Based on the observations of Heald et al., approximately 20% of colorectal adenomatous polyps in *PTEN* mutation carriers developed colorectal adenocarcinoma (19). The result was like the evidence shown by Stanich et al., who diagnosed colorectal cancer in 16% of CS patients (20). However, cases of duodenal carcinoma with CS are rarely reported, let alone in Asian patients (21). So, it is difficult to make a rapid diagnosis when the very first symptom is gastrointestinal bleeding. We assumed that the lesion of the duodenum is the critical point of bleeding because the level of hemoglobin recovered to normal after pancreaticoduodenectomy. In our opinion, routine upper gastrointestinal endoscopy should be considered if bleeding is the first symptom in Asian CS cases (20).

According to the Japanese nationwide multicenter study of CS, the incidence rate of duodenal polyps is 70.2%, which is lower than those of esophageal polyps (85.1%), gastric polyps (91.7%), jejunal/ileal polyps (94.7%), and colorectal polyps (97.7%) (21). They found the incidence is high of colon cancer (6.1% 3 in 49) afflicted with this syndrome. However, it is not known whether patients with Cowden syndrome have a higher risk of upper intestinal cancer (22–24). By reviewing this patient's medical history, we found that the mucosal swelling and rupture near the duodenal papilla were observed by gastroenteroscopy before surgery in 2019, and the pathology suggested severe chronic mucosal inflammation. Therefore, the surgeon did not consider the gastrointestinal bleeding caused by the lesion alteration. During the operation, endoscopy showed that the lower duodenum to the middle and lower parts of the small intestine was full of intestinal polyps of different sizes, and the upper part of the beginning jejunum was about 1.5 m; however, no obvious bleeding point was found, so the proximal 1.5 m small intestine was cut off. According to our experience, if multiple intestinal polyps are found preoperatively, ESD or EGD resection of the tumor and pathological biopsy should be considered. If the lesion is diagnosed as hamartoma, it should be considered CS. Then, the combination of Cowden syndrome should be carefully evaluated according to Cowden diagnostic criteria, and examination and follow-up should be conducted according to the standardized diagnosis and treatment process, which will help early detection of hamartoma malignancy and timely surgical treatment.

There are no clear guidelines for postoperative adjuvant therapy in CS combined with duodenal tumors. According to the NCCN guidelines, the patient's tumor was Stage II A, T2N0M0, so we followed with observation, and there was no apparent recurrence and metastasis for a 1-year follow-up. de Leon et al. reported a duodenal carcinoma (Stage III, T2N1M0) in a 37-year-old man with Cowden/Bannayan syndrome on whom postoperative adjuvant chemotherapy was carried out for 6 months and appeared in good condition within 1-year follow-up (25). Therefore, whether chemotherapy should be performed after surgery needs further study. Our patient's liver function was not well recovered after surgery, but is now back to normal after traditional Chinese medicine treatment.

## Conclusion

Although the specific genetic abnormality that causes Cowden syndrome is presently known as PTEN, duodenum carcinoma with CS is rarely reported, and Asian cases have not been previously described. We report this case to pay more attention to the differential diagnosis of such diseases in the future. It is suggested that if multiple gastrointestinal polyps with gastrointestinal bleeding are encountered, Cowden syndrome should be considered, and timely diagnosis and treatment should be implemented.

## Data Availability

The datasets presented in this article are not readily available because of ethical/privacy restrictions. Requests to access the datasets should be directed to the corresponding author.

## References

[1] Nelen MR Padberg GW Peeters EA Lin AY van den Helm B Frants RR Localization of the gene for Cowden disease to chromosome 10q22-23. Nat Genet. (1996) 13(1):114–6. 10.1038/ng0596-114 8673088

[2] Jornayvaz FR Philippe J. Mucocutaneous papillomatous papules in Cowden’s syndrome. Clin Exp Dermatol. (2008) 33(2):151–3. 10.1111/j.1365-2230.2007.02602.x 18021272

[3] Lloyd KM 2nd Dennis M. Cowden’s disease. A possible new symptom complex with multiple system involvement. Ann Intern Med. (1963) 58:136–42. 10.7326/0003-4819-58-1-136 13931122

[4] Chen CY Chen J He L Stiles BL. PTEN: tumor suppressor and metabolic regulator. Front Endocrinol (Lausanne). (2018) 9:338. 10.3389/fendo.2018.00338 30038596 PMC6046409

[5] Chi SG Kim HJ Park BJ Min HJ Park JH Kim YW Mutational abrogation of the PTEN/MMAC 1 gene in gastrointestinal polyps in patients with Cowden disease. Gastroenterology. (1998) 115(5):1084–9. 10.1016/s0016-5085(98)70078-2 9797362

[6] Olschwang S Serova-Sinilnikova OM Lenoir GM Thomas G. PTEN germ-line mutations in juvenile polyposis coli. Nat Genet. (1998) 18(1):12–4. 10.1038/ng0198-12 9425889

[7] Pilarski R Burt R Kohlman W Pho L Shannon KM Swisher E. Cowden syndrome and the PTEN hamartoma tumor syndrome: systematic review and revised diagnostic criteria. J Natl Cancer Inst. (2013) 105(21):1607–16. 10.1093/jnci/djt277 24136893

[8] Nose V. Genodermatosis affecting the skin and mucosa of the head and neck: clinicopathologic, genetic, and molecular aspect–PTEN-hamartoma tumor syndrome/Cowden syndrome. Head Neck Pathol. (2016) 10(2):131–8. 10.1007/s12105-016-0708-7 26975628 PMC4838969

[9] National Comprehensive Cancer Network: NCCN Clinical Practice Guidelines in Oncology: (NCCN Guidelines): Genetic/familial high-risk assessment: Breast and ovarian (version3.2019). Available at: https://www.nccn.org.

[10] Pilarski R. PTEN hamartoma tumor syndrome: a clinical overview. Cancers (Basel). (2019) 11(6):844. 10.3390/cancers11060844 PMC6627214 31216739

[11] Biller LH Syngal S Yurgelun MB. Recent advances in lynch syndrome. Fam Cancer. (2019) 18(2):211–9. 10.1007/s10689-018-00117-1 30627969 PMC6450737

[12] Toboeva MK Shelygin YA Frolov SA Kuzminov MA Tsukanov AS. MUTYH-associated polyposis. Ter Arkh. (2019) 91(2):97–100. 10.26442/00403660.2019.02.000124 31094179

[13] Buck JL Harned RK Lichtenstein JE Sobin LH. Peutz–Jeghers syndrome. Radiographics. (1992) 12(2):365–78. 10.1148/radiographics.12.2.1561426 1561426

[14] Sandru F Petca A Dumitrascu MC Petca RC Carsote M. Peutz–Jeghers syndrome: skin manifestations and endocrine anomalies. Exp Ther Med. (2021) 22(6):1387. 10.3892/etm.2021.10823 34650635 PMC8506952

[15] De Roock W De Vriendt V Normanno N Ciardiello F Tejpar S. KRAS, BRAF, PIK3CA, and PTEN mutations: implications for targeted therapies in metastatic colorectal cancer. Lancet Oncol. (2011) 12(6):594–603. 10.1016/S1470-2045(10)70209-6 21163703

[16] Pilarski R. Cowden syndrome: a critical review of the clinical literature. J Genet Couns. (2009) 18(1):13–27. 10.1007/s10897-008-9187-7 18972196

[17] Innella G Miccoli S Colussi D Pradella LM Amato LB Zuntini R Colorectal polyposis as a clue to the diagnosis of Cowden syndrome: report of two cases and literature review. Pathol Res Pract. (2021) 218:153339. 10.1016/j.prp.2020.153339 33482532

[18] Schreibman IR Baker M Amos C McGarrity TJ. The hamartomatous polyposis syndromes: a clinical and molecular review. Am J Gastroenterol. (2005) 100(2):476–90. 10.1111/j.1572-0241.2005.40237.x 15667510

[19] Heald B Mester J Rybicki L Orloff MS Burke CA Eng C. Frequent gastrointestinal polyps and colorectal adenocarcinomas in a prospective series of PTEN mutation carriers. Gastroenterology. (2010) 139(6):1927–33. 10.1053/j.gastro.2010.06.061 20600018 PMC3652614

[20] Stanich PP Pilarski R Rock J Frankel WL El-Dika S Meyer MM. Colonic manifestations of PTEN hamartoma tumor syndrome: case series and systematic review. World J Gastroenterol. (2014) 20(7):1833–8. 10.3748/wjg.v20.i7.1833 24587660 PMC3930981

[21] Teramae S Muguruma N Okamoto K Oseto K Nishikawa R Tanoue T Cancer risk and genotype-phenotype correlation in Japanese patients with Cowden syndrome. Int J Clin Oncol. (2022) 27(4):639–47. 10.1007/s10147-022-02116-w 35106660

[22] Carlson GJ Nivatvongs S Snover DC. Colorectal polyps in Cowden’s disease (multiple hamartoma syndrome). Am J Surg Pathol. (1984) 8(10):763–70. 10.1097/00000478-198410000-00005 6496844

[23] Starink TM van der Veen JP Arwert F de Waal LP de Lange GG Gille JJ The Cowden syndrome: a clinical and genetic study in 21 patients. Clin Genet. (1986) 29(3):222–33. 10.1111/j.1399-0004.1986.tb00816.x 3698331

[24] Hamby LS Lee EY Schwartz RW. Parathyroid adenoma and gastric carcinoma as manifestations of Cowden’s disease. Surgery. (1995) 118(1):115–7. 10.1016/s0039-6060(05)80018-2 7604372

[25] de Leon MP Di Gregorio C Giunti L Roncucci L Pedroni M Tinca AC Duodenal carcinoma in a 37-year-old man with Cowden/Bannayan syndrome. Dig Liver Dis. (2013) 45(1):75–8. 10.1016/j.dld.2012.09.017 23117110

